# Heart Failure

**DOI:** 10.1155/2014/730651

**Published:** 2014-02-12

**Authors:** Yanggan Wang, Yanzong Yang, Xun Ai

**Affiliations:** ^1^Department of Cardiology, Zhongnan Hospital, Wuhan University, Wuhan, China; ^2^Department of Cardiology, First Affiliated Hospital of Dalian Medical University, China; ^3^USA Department of Physiology, Loyola University Chicago, Maywood, IL, USA

Heart failure (HF) affects nearly 5 million people in the US and 15 million people worldwide [1, 2]. HF has a considerable impact on affected patients with a high mortality and reduced quality of life. There is also an economic burden to our society due to the huge health-care costs of HF management. To date, the effectiveness of HF diagnosis and management strategies still need to be improved.

When HF is advanced, malfunction of multiple organs is involved and the degree of organ damage is correlated with the progress of HF. Treatment of HF patients depends on the stage of HF. This special issue on HF introduces a new HF evaluation “HLM” system and reviews the role of natriuretic peptide in HF diagnosis and management.

At the end stage of HF, heart transplant often offers the best treatment option. Left ventricular assist devices (LVADs) are surgically implanted pumps that can be used as destination therapy and as a bridge to transplant. However, graft failure is a major cause of mortality among postheart transplant patients. Also, severe mitral regurgitation in HF patients has negative impact on left atrium, right heart, and lung function that ultimately impacts effectiveness of LVAD. The current issue reveals two new approaches, extracorporeal membrane oxygenation rescue and transapical concomitant mitral valve repair to improve the outcome of heart transplant and LVAD therapies. In addition, a review about interaction between renin and IGFII/M6P receptors in nonclassical renin effects and cardiac remodeling sheds light on manipulation of the IGFII/M6P receptor as a potential therapeutic approach to prevent cardiac remodeling in HF. We anticipate more progress in HF management in the near future.

*Yanggan Wang* *Yanggan Wang* *Yanzong Yang* *Yanzong Yang* *Xun Ai* *Xun Ai*
